# Family history of cancer and risk of paediatric and young adult’s testicular cancer: A Norwegian cohort study

**DOI:** 10.1038/s41416-019-0445-2

**Published:** 2019-04-10

**Authors:** Ruby Del Risco Kollerud, Ellen Ruud, Hege S. Haugnes, Lisa A. Cannon-Albright, Magne Thoresen, Per Nafstad, Ljiljana Vlatkovic, Karl Gerhard Blaasaas, Øyvind Næss, Bjørgulf Claussen

**Affiliations:** 1 Institute of Health and Society, University of Oslo, P.O Box 1130 Blindervn, 0318 Oslo, Norway; 2 The National Centre for Occupational Rehabilitation in Norway, Haddlandsvegen 20, 3864 Rauland, Norway; 3 Department of Pediatric Medicine, Oslo University Hospital, Oslo, Norway; 4 Institute of Clinical Medicine, University of Oslo, Oslo, Norway; 5 Department of Oncology, University Hospital of North Norway, Tromsø, Norway; 6 Institute of Clinical Medicine, UIT-The Arctic University, Tromsø, Norway; 7 Department of Internal Medicine, University of Utah School of Medicine, Salt Lake City, UT USA; 8 Department of Biostatistics, Oslo Centre for Biostatistics and Epidemiology, University of Oslo, Tromsø, Norway; 9 Department of Pathology, Oslo University Hospital, Oslo, Norway; 10 Finance Norway, P.O Box 2473, Solli, 0202 Oslo, Norway

**Keywords:** Cancer, Risk factors

## Abstract

**Background:**

The aim of this study was to examine the association of a family history of cancer with the risk of testicular cancer in young adults.

**Methods:**

This is a prospective cohort study including 1,974,287 males born 1951–2015, of whom 2686 were diagnosed with TC before the age of 30.

**Results:**

A history of TC in male relatives was significantly associated with a diagnosis of TC among children and young adults, including brothers (6.3-fold), sons (4.7-fold), fathers (4.4-fold), paternal uncles (2.0-fold) and maternal uncles (1.9-fold). Individuals with a father diagnosed with a carcinoma or sarcoma showed an elevated risk (1.1-fold and 1.8-fold, respectively). A family history of mesothelioma was positively associated with a risk of TC [(father (2.8-fold), mother (4.6-fold) and maternal uncles and aunt (4.4-fold)]. Elevated risks were also observed when siblings were diagnosed with malignant melanoma (1.4-fold). The risk of TC was also increased when fathers (11.1-fold), paternal (4.9-fold) and maternal uncles and aunts (4.6-fold) were diagnosed with malignant neuroepithelial-tumours.

**Conclusion:**

We found an increased risk of TC among children and young adults with a family history of TC, carcinoma, mesothelioma, sarcoma, malignant melanoma and malignant neuroepithelial tumours. Hereditary cancer syndromes might underlie some of the associations reported in this study.

## Background

Germ-cell testicular cancer (TC) is the most common form of cancer in young males in industrialised countries. The global incidence of TC has more than doubled over the past 40 years.^1^ Males born around 1943 and around 1968 in the Nordic countries are at lower risk of TC than men born before and after each of these dates, suggesting a birth-cohort effect in the incidence of TC.^2^

The majority of TC derives from germ cells and can be divided into two major histologic types: pure classic seminoma and non-seminomatous germ cell tumours. These are believed to originate from a common precursor, the germ cell neoplasia in situ (GCNIS).^3^

The aetiology of TC remains unknown. Rapid increases in TC incidence highlight the importance of investigating risk factors involved in the development of this cancer. The strongest risk factors are a family history of the disorder, a previously diagnosed TC, and cryptorchism.^4–9^ Prenatal and postnatal exposure to certain persistent environmental chemicals classified as endocrine disruptors have been reported to be associated with the risk of testicular cancer. However, the evidence is limited.^10^ Socio-economic differences in the incidence rates of TC have also been reported in the Nordic countries.^11^

Cancer in children and young adults generally has more underlying genetic causes compared to cancer in older adults, who have decreased DNA repair capability and longer environmental exposure. In order to identify a young population with a possible inherited cancer predisposition syndrome, an accurate family history integrating information about the site of the origin of the cancer, as well as tumour characteristics such as cancer morphology in relatives, is essential. The most common malignancies associated with hereditary cancer syndromes include morphological types such as sarcomas, carcinomas, epitheliomas, glioblastomas, malignant melanomas and endocrine tumours, as seen in the Li-Fraumeni syndrome, Lynch syndrome, cutaneous malignant melanoma syndrome and multiple endocrine neoplasia.^12^ Consequently, we hypothesise that children and young adults with TC more frequently have relatives with common malignancies associated with hereditary cancer syndromes compared to children without TC.

Previous studies into familial clustering of cancer have typically used organ-specific site classification rather than histological subtypes to examine the risk of TC.^5–9^ Insight into the association of familial clustering of cancers with TC, based on morphological cancer groups, might contribute to the identification of individuals at increased risk of developing the disorder, and might also increase our understanding of this cancer.

The aim of this study was to examine the association of a family history of cancer, evaluating the impact of both morphological groups as well as organ of origin, with the risk of TC in children and young adults. We took advantage of the close to complete population-based registries in Norway that contain uniform and continuously updated information on childbirth, familial relationship, cancer incidence and vital status.

## Materials and methods

### Study population

All males born in Norway from 1951 to 2015 were included in the study. Information on these individuals and their first-degree relatives, uncles and aunts was obtained from the Central Population Register of Norway which has information on the relationship between each individual and his/her relatives.^13,14^ Information on first degree relatives for each individual is almost complete for individuals born in Norway since 1950. We identified cancer among all included males and their relatives through linkage to the Norwegian Cancer Registry using Norwegian personal identification numbers.

Cases or index persons were all males registered in the Norwegian Cancer Registry who had been diagnosed with TC before the age of 30 years between 1951 and 2015. TC was classified into seminomas and non-seminomas using the International Classification of Diseases for Oncology (ICDO-3) and topography (C62).^15^ Unclassified TC (*n* = 271) was excluded from the analyses.

### Cancer among relatives

Morphological groups were classified according to the World Health Organization’s morphologic classification of human cancer into seven main groups: carcinomas, sarcomas, mesotheliomas, tumours of the haematopoietic and lymphoid tissues, Kaposi sarcomas, other specified cancer types, and unspecified types of cancer (Supplementary Table 1).^15,16^ Kaposi sarcomas, some unspecified types of cancer and some morphological subgroups were excluded from the analysis due to small sample sizes.

A separate analysis of the major morphological groups was also conducted using the organ of origin of the cancer in relatives. Analyses of cancer were conducted among relatives based on the anatomic site (topography) of the body in which the cancer originated using the International Statistical Classification of Diseases and related health problems (ICD-10).^17^ To reduce the width of our confidence intervals and to increase precision, ICD-10 codes for which at least two relatives of the index persons diagnosed with cancer were identified were included. This strategy was applied to all the analyses. The following organs and codes were considered: Lip, oral cavity and pharynx (C00-C14), oesophagus (C15), stomach (C16), colon (C18), rectum (C20), pancreas (C25), lung (C34), skin (C43–C44), breast (C50), cervix uteri (C53), Corpus uteri(C54), ovary (C56), prostate (C61), kidney (C64), ureter (C66), bladder (C67) and thyroid (C73).

### Other variables

Families might have different socioeconomic status and levels of environmental exposure that could potentially confound our findings. Models were adjusted for mother or father’s education. Adjustments for the number of family members were also made to account for the difference in family size. Information on parent’s educational level was obtained from Statistics Norway and was categorised into five subgroups according to the International Standard Classification of Education: primary education (<10 years), secondary education and tertiary vocational education (10–14 years), and higher education (equivalent to bachelor, master or PhD).^18^ To control for birth-cohort effects reported in other studies, adjustments were made for year of birth.^2^

### Statistical analysis

Cox proportional hazard regression was used to estimate the association between the incidence of cancer in relatives and risk of TC. Testicular cancer in a child or young adult was the dependent variable and cancer in relatives the explanatory variable. Age was used as time scale. The follow-up period for each child and young adult was from birth to the age of cancer diagnosis, with censoring at the age of 30 years, death, emigration or end of study. All analyses were adjusted for number of relatives (continuous variable), according to which relative was examined.

The risk of TC according to the relationship of the cancer-affected relatives was examined to determine whether there were differences in risk according to kinship. The hazard ratio (HR) and 95% confidence intervals (CI) for morphological types are presented separately for father, mother, siblings, paternal uncles and aunts, and maternal uncles and aunts. Risk estimates for cancer by ICD-10 subtypes included groups of relatives: parents, siblings and both paternal and maternal uncles and aunts. These last analyses were conducted separately for seminomas and non-seminomas. Due to the limited number of cases diagnosed with TC before the age of 15 years, data for the whole population were presented. Offspring of the index person were not included because of the low number of cancer cases found in this group. The proportional hazards assumption was verified by plotting Schoenfeld residuals.

All analyses were performed using SPSS version 25 (IBM, Armonk, NY, USA).

## Results

**Fig. 1:**
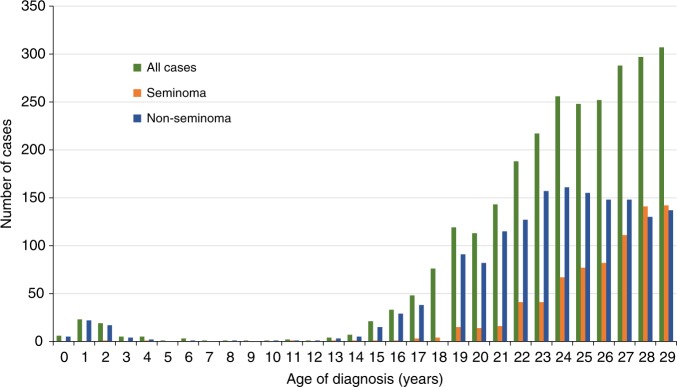
Age distribution of children and young adults <30 years born in Norway during 1951–2015 and who were diagnosed with testicular cancer in the same period

A total of 1,974,287 males born between 1951 and 2015 were followed for risk of TC. During this period 2686 index persons were diagnosed with TC (Table 1). The majority of cases were diagnosed with non-seminomas. Index persons diagnosed with seminomas were slightly older compared with non-seminomas (Table 1; Fig. 1). Most of the study population had information on variables included in the analysis for mother (99.4%) and father (98. 1%).The associations between a family history of cancer and risk of TC were similar before and after adjustment for covariates; adjusted results are reported.

**Table 1:** Descriptive characteristics of a population-based cohort of male children and young adults <30 years born in Norway during 1951–2015

Variable	Cases		Non-cases	
	No.	%	No.	%
	Total = 2686	0.1	1,971,601	99.9
*Tumour histology*				
Seminoma	761	28.3		
Non-seminoma	1654	61.6		
Unclassified	271	10.1		
*Mean age at cancer diagnosis (SD)*				
All cases	24 (4.9)			
Seminoma	26 (3.1)			
Non-seminoma	23 (5.2)			
*Mother’s education (years)*				
<10	919	34.2	267,137	28.8
10–14	1264	47.1	700,203	35.5
>14	474	17.6	395,912	20.1
Missing	29	1.1	308,349	15.6
*Father’s education (years)*				
<10	726	27.0	475,844	24.1
10–14	1332	49.6	783,268	39.7
>14	560	20.8	377,921	19.2
Missing	68	2.5	334,568	17.0
Mean age at cancer diagnosis of the relatives (SD)		Mean (SD)		Mean (SD)
Father	668	64.1 (12.4)	338,567	66.1 (13.1)
Mother	538	61.2 (13.9)	288,954	61.7 (14.9)
Brothers	166	39.7 (16.0)	64,941	47.1 (16.9)
Sisters	121	43.4 (13.5)	70,820	45.4 (14.3)
Sons	14	17.6 (8.5)	5261	18.6 (11.5)
Daughters	8	14.0 (10.8)	5102	20.8 (12.0)
Paternal uncles	221	59.8 (14.3)	120,627	57.9 (15.3)
Paternal aunts	194	54.8 (13.5)	109,509	53.2 (14.4)
Maternal uncles	240	57.3 (13.5)	107,971	56.3 (15.6)
Maternal aunts	204	54.7 (12.0)	103,467	52.1 (14.4)

*SD* standard deviation

### Cancer family history across morphological groups

Associations of risk of TC with a family history of cancer (all cancer subtypes) were observed for multiple relationships and morphological groups (Table 2). Statistically significant associations were observed for index persons where fathers and maternal uncles and aunts were diagnosed with carcinomas HR = 1.11 (1.00–1.23) and HR = 1.15 (1.02–1.30), respectively, particularly the subtype adenocarcinoma. The risk of TC was also elevated when fathers were diagnosed with sarcomas HR = 1.81 (1.10–2.96).

**Table 2:** Adjusted hazard ratios (95% confidence interval) for family history of cancers by morphologic groups and risk of testis cancer among children and young adults <30 years born in Norway during 1951–2015

		Father		Mother		Siblings		Fathers siblings		Mothers siblings
Morphologic groups in relatives	*n*	HR (95% CI)	*n*	HR (95% CI)	*n*	HR (95% CI)	*n*	HR (95% CI)	*n*	HR (95% CI)
Carcinomas	496	**1.11 (1.00–1.23)**	416	1.10 (0.98–1.22)	119	0.91 (0.75–1.11)	311	0.98 (0.86–1.11)	343	**1.15 (1.02–1.30)**
Squamous cell carcinoma	67	1.15 **(**0.90–1.47)	55	1.14 (0.87–1.50)	16	0.91 (0.55–1.49)	26	0.63 (0.43–0.93)	46	1.17 (0.87–1.57)
Urothelial carcinoma	39	1.06 **(**0.77–1.46)	14	1.29 (0.76–2.19)	4	0.76 (0.29–2.04)	15	0.87 (0.52–1.45)	18	1.11 (0.70–1.77)
Adenocarcinoma	343	**1.13 (1.01–1.27)**	295	1.04 (0.92–1.18)	97	1.00 (0.81–1.23)	238	1.04 (0.91–1.20)	250	**1.20 (1.05–1.37)**
Other specific carcinomas	35	1.28 **(**0.91–1.79)	31	1.32 (0.93–1.89)	6	0.64 (0.29–1.44)	19	0.90 (0.57–1.41)	25	1.18 (0.79–1.75)
Neuroendocrine carcinoma	7	1.41 **(**0.67–2.97)		1.70 (0.85–3.40)	5	1.72 (0.71–4.13)	4	0.86 (0.32–2.29)	4	0.84 (0.31–2.24)
Sarcomas and soft tissue tumours	16	**1.81 (1.10–2.96)**	12	1.36 (0.77–2.39)	8	0.95 (0.47–1.90)	11	1.11 (0.61–2.01)	6	0.60 (0.27–1.34)
Mesothelioma	8	**2.77 (1.38–5.56)**	2	**4.62 (1.16–18.50)**	–	NC	–	NC	5	**4.44 (1.85–10.70)**
Tumours of hematopoietic and lymphoid tissues	50	0.99 **(**0.75–1.31)	37	1.00 (0.72–1.38)	24	0.99 (0.66–1.48)	45	1.17 (0.87–1.58)	41	1.13 (0.83–1.54)
Myeloid	5	0.57 **(**0.24–1.38)	5	0.74 (0.31–1.77)	2	0.45 (0.11–1.82)	4	0.60 (0.23–1.61)	8	1.19 (0.60–2.39)
B-cell neoplasms	34	1.17 **(**0.83–1.64)	26	1.19 (0.80–1.75)	15	1.58 (0.95–2.62)	28	1.29 (0.89–1.87)	21	0.99 (0.65–1.53)
Non-Hodgkin lymphoma	12	0.87 **(**0.49–1.53)	17	1.49 (0.93–2.41)	8	1.45 (0.72–2.90)	17	1.41 (0.88–2.28)	12	1.04 (0.59–1.83)
Multiple myeloma and other plasma cell	12	1.32 **(**0.75–2.33)	5	0.77 (0.32–1.85)	3	1.59 (0.51–4.95)	7	1.23 (0.59–2.58)	5	0.93 (0.39–2.25)
Hodgkin lymphoma	4	1.42 **(**0.53–3.80)	–	NC	2	0.46 (0.12–1.86)	4	1.30 (0.49–3.48)	3	0.90 (0.29–2.79)
Malignant melanoma	21	0.66 **(**0.43–1.01)	35	1.08 (0.77–1.50)	31	**1.45 (1.01–2.06)**	23	0.75 (0.50–1.14)	37	1.22 (0.88–1.69)
Seminoma (C62)	22	**4.45 (2.92–6.77)**	–	NC	25	**3.75 (2.52–5.56)**	8	1.89 (0.94–3.78)	6	1.20 (0.54–2.67)
Non-seminoma (C62)	10	**4.50 (2.42–8.37)**	–	NC	46	**8.24 (6.15–11.04)**	6	**2.37 (1.06–5.30)**	8	**2.87** (**1.43–5.76)**
Gliomas (C71)	4	0.41 **(**0.15–1.10)	6	0.90 (0.40–2.01)	9	1.14 (0.59–2.19)	5	0.57 (0.24–1.38)	3	0.35 (0.11–1.07)
Meningiomas	4	1.12 **(**0.42–2.99)	11	1.12 (0.62–2.02)	4	1.04 (0.39–2.77)	4	0.61 (0.24–1.64)	5	0.79 (0.33–1.90)
Malignant neuroepithelial tumours	2	**11.15 (2.78**–**44.57)**	–	NC	2	1.61 (0.40–6.45)	2	**4.92 (1.23–19.70)**	2	**4.62** **(1.15–18.50)**

The model was adjusted for child’s birth year and number of relatives according to type analysis. The model for parents and sibling was also adjusted for mother or father’s education

Cases: number of cancer cases with relatives affected

NC: children with <2 relatives diagnosed with cancer estimates were no calculated. We included morphologic groups were we find at least 2 relatives of the index persons diagnosed with cancer

The bold values mean Hazard ratios statistically significant at 5% level

A family history of mesothelioma was associated with the risk of TC. The risk increased when father, mother, or maternal uncles and aunts of index persons were diagnosed with mesothelioma HR = 2.77 (1.38–5.56), HR = 4.62 (1.16–18.50) and HR = 4.44 (1.85–10.70), respectively.

**Fig. 2:**
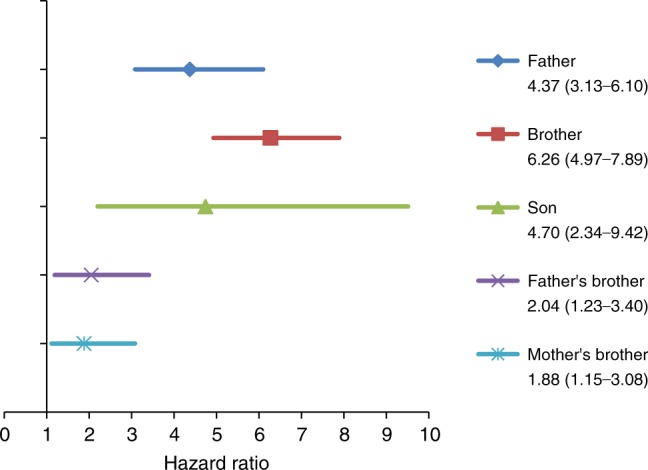
Adjusted hazard ratios (95% confidence interval) for the association of family history of testicular cancer in first-degree relative’s males and uncles with risk of testicular cancer in children and young adults <30 years born in Norway during 1951–2015. The model was adjusted for child’s birth year and number of relatives according to type analysis. The model for father and brothers was also adjusted for mother or father’s education

Overall, the risk of TC was associated with a family history of TC in male relatives (Fig. 2). The highest risk was noted among index persons with a brother with TC (6-fold increased risk), followed by individuals with a son or father diagnosed with testis cancer (4-fold increased risk). Statistically significant results were also observed for paternal and maternal uncles (Fig. 2). The highest risk was observed among index persons with brothers diagnosed with TC type non-seminomas HR = 8.24 (6.15–11.04). Among siblings the risk was also elevated for malignant melanoma HR = 1.45 (1.01–2.06).

A positive family history of malignant neuroepithelial tumours was also associated with an increased risk of TC. The elevated risk was observed among index person with an affected father and paternal and maternal uncles and aunts HR = 11.15 (2.78–44.57); HR = 4.92 (1.23–19.70) and HR = 4.62 (1.15–18.50), respectively.

### Cancer family history of cancer by cancer site

The risk of developing TC among index persons increased overall when parents were diagnosed with squamous cell carcinoma of the lung (Table 3) and significantly increased for non-seminomas HR = 1.58 (1.01–2.46), but not significantly for seminomas. A maternal diagnosis of cervical cancer increased the risk of seminoma among index persons HR 1.92 (1.00–3.72). Index persons with a sister diagnosed with breast cancer of type adenocarcinoma had an elevated risk of TC HR = 2.00 (1.24–3.21).

**Table 3:** Adjusted hazard ratios (95% confidence interval) for family history of carcinomas by the most common subtypes among parents, siblings, uncles and aunts, and risk of testis cancer among children and young adults <30 years born in Norway during 1951–2015

	Parents						Siblings					
	All cases		Seminoma		Non-seminoma		All cases		Seminoma		Non-seminoma	
Type carcinoma in relatives	*n*	HR (95% CI)	*n*	HR (95% CI)	*n*	HR (95% CI)	*n*	HR (95% CI)	*n*	HR (95% CI)	*n*	HR (95% CI)
*Squamous cell carcinoma*												
Lips, oral cavity and pharynx	14	0.93 (0.55–1.57)	4	0.93 (0.35–2.48)	10	1.14 (0.61–2.12)	–	NC	–	NC	–	NC
Lung	32	**1.41** (**1.00–2.00)**	7	1.09 (0.51–2.29)	20	**1.58 (1.01–2.46)**	–	NC	–	NC	–	NC
Skin	35	1.00 (0.72–1.42)	9	0.89 (0.46–1.72)	23	1.19 (0.78–1.79)	4	1.41 (0.53–3.77)	–	NC	–	NC
Cervix uteri	21	1.26 (0.82–1.94)	9	**1.92 (1.00–3.72)**	10	1.01 (0.54–1.88)	6	0.89 (0.40–1.98)	3	1.59 (0.51–4.94)	3	0.78 (0.25–2.43)
*Urothelial carcinoma*												
Ureter	2	1.48 (0.37–5.91)	–	NC	2	2.69 (0.67–10.8)	–	NC	–	NC	–	NC
Bladder	47	1.10 (0.83–1.48)	10	0.82 (0.44–1.53)	30	1.25 (0.87–1.79)	4	0.86 (0.32–2.29)	–	NC	–	NC
*Adenocarcinoma*												
Oesophagus	6	1.60 (0.72–3.56)	2	1.84 (0.46–7.37)	3	1.36 (0.44–4.23)	–	NC	–	NC	–	NC
Stomach	26	1.12 (0.76–1.65)	10	1.53 (0.82–2.87)	12	0.93 (0.52–1.64)	–	NC	–	NC	–	NC
Colon	91	1.10 (0.89–1.35)	28	1.16 (0.79–1.70)	50	1.05 (0.79–1.40)	10	1.01 (0.54–1.88)	2	0.73 (0.18–2.85)	6	1.13 (0.51–2.53)
Rectum	45	1.08 (0.88–1.34)	15	1.25 (0.75–2.10)	24	1.02 (0.68–1.52)	9	1.50 (0.78–2.90)	2	1.23 (0.31–4.93)	5	1.55 (0.64–3.73)
Pancreas	23	1.25 (0.83–1.90)	4	0.75 (0.28–2.00)	14	1.34 (0.79–2.27)	2	0.89 (0.22–3.55)	–	NC	–	NC
Lung	31	0.97 (0.68–1.38)	8	0.86 (0.43–1.73)	16	0.86 (0.53–1.42)	5	1.08 (0.45–2.59)	2	1.60 (0.40–6.45)	2	0.81 (0.20–3.24)
Breast (female)	123	1.04 (0.87–1.25)	27	0.78 (0.53–1.14)	72	1.02 (0.81–1.30)	42	1.28 (0.94–1.75)	18	**2.00 (1.24–3.21)**	18	1.01 (0.63–1.60)
Cervix uteri	2	0.77 (0.19–3.08)	–	NC	2	1.26 (0.31–5.04)	–	NC	–	NC	0	NC
Corpus uteri	21	0.92 (0.60–1.41)	10	1.48 (0.79–2.77)	6	0.46 (0.21–1.02)	–	NC	–	NC	–	NC
Ovary	19	1.05 (0.67–1.66)	4	0.76 (0.28–2.04)	11	1.06 (0.59–1.92)	4	1.09 (0.41–2.91)	3	2.97 (0.95–8.99)	–	NC
Prostate	179	1.14 (0.97–1.33)	56	1.21 (0.92–1.60)	98	1.08 (0.88–1.33)	16	0.94 (0.57–1.54)	5	1.09 (0.45–2.56)	7	0.79 (0.37–1.67)
Kidney	26	1.10 (0.75–1.62)	7	1.03 (0.49–2.16)	14	1.01 (0.60–1.72)	3	0.59 (0.19–1.85)	–	NC	2	0.73 (0.18–2.93)
Thyroid	12	1.25 (0.71–2.20)	4	1.45 (0.54–3.87)	7	1.19 (0.57–2.49)	3	0.68 (0.22–2.12)	–	NC	2	0.80 (0.20–3.21)
No carcinomas	2399	0.88 (0.78–1.00)	674	0.83 (0.66–1.05)	1497	0.94 (0.80–1.11)	158	**1.87 (1.59–2.21)**	44	**1.85 (1.36–2.52)**	94	**1.95 (1.58–2.41)**

The model was adjusted for child’s birth year and number of relatives according to type analysis. The model for parents and sibling was also adjusted for mother or father’s education

N, number of cancer cases with relatives affected

NC, children with <2 relatives diagnosed with cancer estimates were no calculated. We included the ICD-10 codes were we find at least 2 relatives of the index persons diagnosed with cancer

The bold values mean Hazard ratios statistically significant at 5% level

A positive family history of urothelial carcinoma among uncles and aunts was associated with an elevated risk of TC in index persons HR = 3.15 (1.01–9.81) (Supplementary Table 2). Cancer of other organs showing elevated risk of TC was found among index persons with uncles and aunts diagnosed with stomach cancer of type adenocarcinoma HR = 2.38 (1.14–4.95) (Supplementary Table 2).

Elevated risk was also observed in index persons with siblings affected by other types of cancer, excluding carcinomas HR = 1.87 (1.59–2.21).

## Discussion

This population-based cohort study suggests that there is a significantly elevated risk of TC among individuals with a family history of cancer that extends beyond TC. Elevated risks were observed for family history of carcinoma, mesothelioma, sarcomas, testicular germ tumours, malignant melanoma and malignant neuroepithelial tumours. Such findings of elevated risks among these morphological groups, with the exception of germ cell tumours and melanoma in a large population study, have not been previously reported.

An increased risk of developing TC in males with first-degree relatives diagnosed with TC is consistent with previous studies.^5–9^ We also found an elevated risk of TC among index persons with paternal and maternal uncles diagnosed with TC (Fig. 2). A recent study reported elevated risk of TC with a family history of breast cancer, melanoma, lung cancer and cancer in the central nervous system.^19^

Human testicular cancer susceptibility genes have not yet been identified. The putative gene mapped to *Xq27* is postulated to confer an increased risk of TC as well as cryptorchism.^20^ Cancer is recognised as a disease that result from gradual accumulation of somatic mutations in the genome.^21^ The mutation frequency in the whole genome between generations of humans (parent to child) is about 70 new mutations per generation.^22^ Carcinomas, however, have much higher mutation frequencies.^23^ The high mutation frequencies in carcinomas reflect the genome instability characteristic of cancer. New results from the Cancer Genome Atlas Research Project have identified genetic mutations that are common among 12 different types of cancer, including carcinomas, adenocarcinomas and melanomas. This reflects the growing understanding that tumours can be defined by their underlying biology rather than their location in the body.^24^ Our results provide evidence of an increased familial risk of TC associated with a general family history of cancer; especially perhaps subtypes squamous cell carcinoma, urothelial carcinoma and adenocarcinoma. In an analysis of carcinomas by organ site, elevated risks were found for the lung, breast, cervix, ureter and stomach. Squamous cell carcinoma of the lung accounts for around 30% of all lung cancer. Although this histological subtype has a stronger association with smoking than any other type of lung cancer, family history and exposure to asbestos or radon are also risk factors for this type of cancer.

The association between a family history of mesothelioma and TC in young TC patients has not been previously reported. A particularly striking finding in the present study was the significant increase in the risk of TC among index persons with parents and uncles and aunts with mesothelioma. The increased risk was consequent for both seminoma and non-seminoma. However, the analyses are based on a small number of cases. Travis and colleagues reported statistically significantly increased risk of malignant mesothelioma (3.4-fold) in TC survivors.^25^ They concluded that the treatment of cancer patients with very high doses of radiation, or the impact of the natural history of the disease might explain the observed excess risk.

Heritable mutations in the *BAP1* tumour suppressor gene predispose individuals to mesothelioma and other forms of cancer. The *BAP1* tumour predisposition syndrome is a novel cancer syndrome characterised by onset at an early age of melanomas and, later in life, by a high incidence of mesothelioma, melanoma and renal cell carcinoma.^26–29^ The full spectrum of this syndrome is still being characterised through the discovery of new associated tumours, including cutaneous squamous cell carcinoma,^30^ basal cell carcinoma,^31,32^ lung adenocarcinoma,^33^ oesophageal adenocarcinoma,^34^ breast cancer,^35^ rhabdoid meningioma,^36^ neuroendocrine tumours,^37^ and certain types of sarcoma.^38,39^ Some types of cancer associated with this syndrome may also have a poor prognosis. In the present study, significant associations of TC were identified with a cancer family history of cancers related to the *BAP1* tumour predisposition syndrome, including mesothelioma, melanoma, squamous cell carcinomas, adenocarcinomas, sarcomas and breast cancer.

Excess familial risk was also identified in other morphological groups, sarcomas, malignant melanoma, testicular germ cell tumours and malignant neuroepithelial tumours. Familial clustering of two or more cancer sites is usually attributed to specific, rare and dominantly inherited susceptibility genes. The increased risk of TC we found in index persons with male relatives with TC would support this theory. Some families are afflicted by a well-known rare inherited syndrome which frequently includes sarcomas, for example, as seen in the Li-Fraumeni syndrome. Hereditary melanomas can appear as part of a Familial Melanoma Syndrome or a Mixed Cancer Syndrome.^40^ Usually this occurs by mutations in the *CDKN2A* gen. This syndrome not only increases the risk of melanoma but also other malignancies, such as sarcomas, lymphomas, cancer of the pancreas, lung, breast, cervix, ovary, stomach colon, brain and urinary bladder.

Retinoblastoma, neuroblastoma, ganglioglioma and neuroepithelioma are among the most common neuro-epitheliomatous neoplasms. These cancers are associated with several hereditary cancer syndromes such as hereditary retinoblastoma, where sarcomas are the most frequent second cancer. Additional cancers found in this syndrome include leukaemia, lymphoma, melanoma, lung and bladder cancer.^14^ Neuroblastoma is also a feature of neurofibromatosis type 1 and the Beckwith-Wiedemann syndrome.

The results of the present study taken together with the results of other studies suggest that hereditary cancer syndromes could be involved in a predisposition to TC in young males. However, these findings do not exclude a possible influence of shared environmental factors among family members. Further research is needed to clarify the molecular genetic basis of testicular cancer and to identify potential susceptibility genes playing a role in the aetiology of the disease. More research is also needed to assess the possible interplay between environmental factors and genetic susceptibility in cancer causation. A better understanding of testicular cancer predisposition and biology will lead to further refinements in the clinical management of the disease, especially regarding identification of individuals of higher risk.

The present study has several strengths. It is based on all births in Norway from 1951 to 2015. Thus, bias caused by a skewed study sample is unlikely. Information on the variables of interest was obtained from the linkage of national population-based registers (removing ascertainment and recall bias), and the high quality of cancer case registration permitted a complete follow-up of the study population. Data from the Norwegian Cancer Registry is considered reasonably accurate and close-to-complete.^41^

The study also has some limitations. The present study is a registry-based study, with no access to biological samples for genetic analyses. Information on cancer risk among parents at a young age in the first birth cohort period was not available and could introduce bias by left truncation. The follow-up for cancer among relatives was until 2015. This could introduce bias. Parents and other first-degree relatives of children and young adults are often young, and cancer may not have developed yet. As noted, the number of cancer cases observed among the offspring of index cases was insufficient to include these relationships in the study. However, both of these sources of bias would lead to an underestimation of the actual cancer risk. Although this is a large cohort study some of these associations were based on small numbers that might lead to some false positive results. Thus, the results are best considered together with those from similar studies. The study is based on children from Norway, primarily a white population. It is unclear whether these data can be generalised to non-Caucasian populations. Finally, this study should be considered to be hypothesis generating due to the high number of hypotheses considered.

## Conclusion

We found significantly elevated risks of TC among children and young adults with a family history of testicular cancer, carcinomas, mesothelioma, sarcomas, malignant melanoma and malignant neuroepithelial tumours.

Our results show that many of the cancers identified in the relatives of TC cases are found to be associated with the spectrum of several known cancer syndromes, such as cancers observed in *BAP1* tumour predisposition syndrome, Li-Fraumeni syndrome, Familial Melanoma Syndrome, neurofibromatosis and hereditary retinoblastoma. Other syndromes cannot be excluded. However, the present study contains multiple comparisons and the results must be interpreted with caution. Further research into the genetic and environmental interactions associated with the risk of TC is critically important.

## Supplementary information

Supplementary Tables

## Data Availability

All data generated or analysed during this study are included in this published article and its supplementary information files.
