# Supplementary material for: Family history of cancer and risk of paediatric and young adult’s testicular cancer: A Norwegian cohort study
Source: Br J Cancer. 2019 Apr 10;120(10):1007–14. doi: 10.1038/s41416-019-0445-2 (PMC6734662; doi:10.1038/s41416-019-0445-2)
Supplement: Supplementary file 1 — Supplementary Tables [file 41416_2019_445_MOESM1_ESM.docx]

**Supplementary Table 1. Groups of malignant neoplasms considered to be histologically ‘different’ for the purpose of defining multiple tumours** (adapted from Berg JW. Morphologic classification of human cancer. In: Schottenfeld D & Fraumeni JF Jr. Cancer Epidemiology and Prevention, 2^nd^ edition, Chapter 3 of Section 1: Basic Concepts. Oxford, New York, Oxford University Press, pp. 28-44)

| **Group** | **ICD-O-3 Morphology** |
| --- | --- |
| Carcinomas |  |
| Squamous | 8051-8084 |
| Transitional or urothelial carcinoma | 8120-8131 |
| Basal cell carcinomas | 8090-8110 |
| Adenocarcinomas | 8140-8149, 8160-8162, 8190-8221, 8260-8337, 8350-8551, 8570-8576, 8940-8941 |
| Other specific carcinomas | 8030-8046, 8150-8157, 8170-8180, 8230-8255, 8340-8347, 8560-8562, 8580-8671 |
| Neuroendocrine tumours | 8150-8153 (pancreas), 8155-8156 (vipoma), 8240-8242 (carcinoid), 8246 (nevoid carcinoma), 8249 (atypical carcinoid) |
| Sex cord-stromal tumours | (C62) - 8591, 8592, 8600, 8620, 8622, 8640, 8642, 8643, 8650 |
| Unspecified carcinomas (NOS) | 8010-8015, 8020-8022, 8050 |
| Sarcomas and soft tissue tumours | 8680-8713, 8800-8921, 8990-8991, 9040-9044, 9120-9125, 9130-9136, 9141-9252, 9370-9373, 9540-9582 |
| Mesothelioma | 9050-9055 |
| Tumours of hematopoietic and lymphoid tissues |  |
| Myeloid | 9840, 9861-9931, 9945-9946, 9950, 9961-9964, 9980-9987 |
| B-cell neoplasms | 9670-9699, 9728, 9731-9734, 9761-9767, 9769, 9823-9826, 9833, 9836, 9940 |
| Non-Hodgkin lymphoma | 9670-9699 |
| Multiple myeloma and other plasma cell | 9731-9734 |
| T-cell and NK-cell neoplasms | 9700-9719, 9729, 9768, 9827-9831, 9834, 9837, 9948 |
| Hodgkin lymphoma | 9650-9667 |
| Mast-cell tumours | 9740-9742 |
| Histiocytes and accessory lymphoid cells | 9750-9758 |
| Unspecified types | 9590-9591, 9596, 9727, 9760, 9800-9801, 9805, 9820, 9832, 9835, 9860, 9960, 9970, 9975, 9989 |
| Kaposi sarcoma | 9140 |
| Other specified types of cancer | 8720-8790, 8930-8936, 8950-8983, 9000-9030, 9060-9110, 9260-9365, 9380- 9539 |
| Malignant melanoma | 8720-8780 |
| Complex mixed and stromal neoplasms | 8930-8936 |
| Nefroblast-hepatoblast-others | 8950-8983 |
| Fibro epithelial neoplasm | 9000-9030 |
| Germ cell tumours | 9060-9103 |
| Seminoma (C62) | 9061-9062(C62) |
| Non-seminoma (C62) | 9070, 9071, 9080, 9081, 9084, 9085, 9100, 9101(C62) |
| Gliomas (C71) | 9380-9480 (C71) |
| Meningiomas | 9530-9539 |
| Malignant neuroepithelial tumours | 9490-9523 |
| Unspecified types of cancer | 8000-8005 |

Source: World Health Organization. International Classification of Diseases for Oncology, Third Edition, First Revision. Geneva: World Health Organization, 2013. ISBN 978 92 4 154849 6.

Seminoma testicular cancers were identified with morphology codes: 9061 (seminoma, not otherwise specified (NOS)) and 9062 (seminoma, anaplastic). Non-seminomas (embryonal carcinoma, yolk sac carcinoma, choriocarcinoma, teratoma and mixed) were identified with morphology codes: 9070, 9071, 9080, 9081, 9084, 9085, 9100 and 9101.

**Supplementary Table 2. Adjusted hazard ratios (95% confidence interval) for family history of carcinomas by the most common subtypes among uncles and aunts, and risk of testis cancer among children and young adults <30 years born in Norway during 1951-2015.**

|  | **Maternal and paternal uncles and aunts** | | | | | |
| --- | --- | --- | --- | --- | --- | --- |
|  | **All cases** | | **Seminoma** | | **Non-seminoma** | |
| **Type carcinoma in relatives** | **N** | **HR (95% CI)** | **N** | **HR (95% CI)** | **N** | **HR (95% CI)** |
| *Squamous cell carcinoma* |  |  |  |  |  |  |
| Lips, oral cavity and pharynx | 15 | 1.06 (0.63-1.79) | 3 | 0.79 (0.25-2.49) | 11 | 1.15 (0.62-2.14) |
| Lung | 16 | 1.08 (0.65-1.79) | 3 | 0.77 (0.24-2.38) | 12 | 1.23 (0.68-2.21) |
| Skin | 22 | 1.11 (0.72-1.73) | 7 | 1.35 (0.62-2.97) | 11 | 0.84 (0.46-1.59) |
| Cervix uteri | 10 | 0.64 (0.34-1.20) | 4 | 0.99 (0.36-2.71) | 5 | 0.46 (0.19-1.12) |
| *Urothelial carcinoma* |  |  |  |  |  |  |
| Ureter | 3 | **3.15 (1.01-9.81)** | - | NC | 3 | **4.72 (1.51-14.77)** |
| Bladder | 27 | 0.95 (0.64-1.41) | 7 | 0.93 (0.42-1.99) | 18 | 0.95 (0.59-1.56) |
| *Adenocarcinoma* |  |  |  |  |  |  |
| Esophagus | 4 | 1.21 (0.45-3.23) | 2 | 2.28 (0.56-9.19) | 2 | 0.90 (0.22-3.62) |
| Stomach | 18 | 1.38 (0.86-2.23) | 8 | **2.38 (1.14-4.95)** | 9 | 1.03 (0.54-2.05) |
| Colon | 58 | 1.04 (0.78-1.39) | 10 | 0.67 (0.34-1.31) | 46 | 1.24 (0.90-1.75) |
| Rectum | 33 | 1.10 (0.76-1.58) | 6 | 0.76 (0.33-1.74) | 25 | 1.23 (0.81-1.89) |
| Pancreas | 15 | 1.17 (0.69-1.97) | 6 | 1.75 (0.76-4.06) | 8 | 0.93 (0.46-1.89) |
| Lung | 27 | 1.06 (0.70-1.57) | 6 | 0.87 (0.38-2.01) | 19 | 1.11 (0.69-1.78) |
| Breast (female) | 129 | 1.21 (0.96-1.50) | 31 | 1.10 (0.70-1.72) | 95 | 1.29 (0.99-1.69) |
| Cervix uteri | 5 | 1.74 (0.72-4.23) | - | NC | 3 | 1.51 (0.48-4.73) |
| Corpus uteri | 23 | 1.26 (0.82-1.94) | 6 | 1.24 (0.53-2.88) | 15 | 1.21 (0.71-2.06) |
| Ovary | 16 | 1.10 (0.66-1.82) | 5 | 1.30 (0.52-3.21) | 9 | 0.90 (0.46-1.77) |
| Prostate | 133 | 1.16 (0.93-1.44) | 35 | 1.13 (0.73-1.74) | 89 | 1.17 (0.89-1.53) |
| Kidney | 20 | 0.95 (0.60-1.51) | 8 | 1.47 (0.70-3.04) | 11 | 0.77 (0.42-1.43) |
| Thyroid | 14 | 1.35 (0.79-2.31) | 2 | 0.73 (0.18-3.02) | 12 | 1.67 (0.93-3.03) |
| No carcinomas | 2090 | 1.06 (0.88-1.28) | 605 | 1.12 (0.78-1.62) | 1251 | 1.01 (0.80-1.26) |

The model was adjusted for child’s birth year and number of relatives according to type analysis.

N: number of cancer cases with relatives affected.

NC: children with <2 relatives diagnosed with cancer estimates were no calculated. We included the ICD-10 codes were we find at least 2 relatives of the index persons diagnosed with cancer.
